# Cardiac Involvement in Emery–Dreifuss Muscular Dystrophy, from Arrhythmias to Heart Failure and Sudden Death: A Contemporary Review

**DOI:** 10.3390/jcm15093286

**Published:** 2026-04-25

**Authors:** Lucio Giuseppe Granata, Maria Claudia Lo Nigro, Fabiana Cipolla, Nicola Ferrara, Anna Rosa Napoli, Marcello Marchetta, Simona Giubilato, Pasquale Crea, Giuseppe Dattilo, Olimpia Trio, Giuseppe Andò, Cesare de Gregorio, Giuseppina Maura Francese

**Affiliations:** 1 Cardiology Division, Garibaldi-Nesima Hospital, ARNAS Garibaldi, 95122 Catania, Italy; 2 Cardiology Unit, Department of Clinical and Experimental Medicine, University Hospital “G. Martino”, University of Messina, 98125 Messina, Italy; mariaclaudialonigro@gmail.com (M.C.L.N.); fabiana.cipolla@libero.it (F.C.); annarosanapoli1510@gmail.com (A.R.N.); pcrea@unime.it (P.C.); giuseppe.dattilo@unime.it (G.D.); cesare.degregorio@gmail.com (C.d.G.); 3 Cardiology Division, Barone Lombardo Hospital, 92024 Canicattì, Italy; nicolaferrara96@virgilio.it; 4 Cardiology Department, Policlinico Tor Vergata, 00133 Rome, Italy; marcello.marchetta1997@gmail.com; 5 Cardiology Division, Cannizzaro Emergency Hospital, 95126 Catania, Italy; simogiub@hotmail.com; 6 Cardiology Department, Azienda Ospedaliera Papardo, 98158 Messina, Italy; giuseppeando1975@gmail.com; 7 Department of Clinical and Experimental Medicine, University of Messina, 98158 Messina, Italy

**Keywords:** Emery–Dreifuss muscular dystrophy, nuclear envelope diseases, cardiomyopathy, cardiac conduction disorders, atrial fibrillation, heart failure, ventricular arrhythmias, sudden cardiac death, implantable cardioverter–defibrillator

## Abstract

Emery–Dreifuss muscular dystrophy (EDMD) is a rare inherited neuromuscular disorder within the spectrum of nuclear envelope diseases, classically characterized by early musculo-tendinous contractures, slowly progressive myopathy, and cardiac involvement dominated by conduction disease and arrhythmias, with variable evolution toward cardiomyopathy and heart failure. This narrative review provides a comprehensive and clinically actionable synthesis of cardiovascular manifestations across EDMD genotypes and phenotypes, outlining pragmatic diagnostic and therapeutic pathways for real-world care. A targeted literature search was performed in PubMed, Embase, and Web of Science, focusing on studies addressing cardiovascular involvement in EDMD. Relevant original studies, case series, registries, guideline documents, and high-quality reviews were selected and synthesized narratively, with particular emphasis on diagnostic strategies, risk stratification, and management approaches. Cardiac involvement in EDMD encompasses a broad and heterogeneous spectrum, including atrial disease and conduction disturbances, ventricular arrhythmias, dilated cardiomyopathy, thromboembolic complications, and sudden cardiac death. Phenotypic expression varies according to the underlying genetic substrate, with distinct atrial- and ventricular-dominant trajectories. Early recognition and structured cardiovascular surveillance are essential to guide timely intervention, including anticoagulation, device therapy, and heart failure management. Despite growing awareness, significant gaps remain in risk prediction and standardized management strategies. EDMD represents a paradigmatic model of cardiomyopathy characterized by prominent electrical instability and systemic involvement. A structured, genotype- and phenotype-informed approach centered on early surveillance, proactive arrhythmia and thromboembolic risk management and timely device therapy may improve clinical decision-making in real-world settings. Future perspectives include the integration of precision medicine and the development of gene- and pathway-targeted therapies, with the potential to shift from symptomatic management toward disease-modifying strategies.

## 1. Introduction

### 1.1. Emery–Dreifuss Muscular Dystrophy: Disease Overview

In 1966, the neurologists Alan E. Emery and Fritz E. Dreifuss first identified a clinical phenotype distinct from other progressive muscular dystrophies. In their original report, Emery and Dreifuss described a benign X-linked neuromuscular phenotype characterized by a distinctive clinical triad [1]:

- Early contractures (particularly involving the elbows, Achilles tendons, and cervical spine), often preceding the onset of muscle weakness;
- Slowly progressive myopathy with a scapulohumeral and peroneal distribution;
- Cardiac involvement with conduction system disease, responsible for syncope and sudden cardiac death, already recognized as an integral component of the disorder.

Since the 1960s, the term “Emery–Dreifuss syndrome” has been used to describe a group of rare inherited disorders sharing muscular, joint, and cardiac manifestations caused by different genetic mutations. These conditions are classified as neuromuscular degenerative disorders, similarly to Duchenne muscular dystrophy and Becker muscular dystrophy, but belonging to the spectrum of nuclear envelope diseases and generally associated with a more favorable clinical course [2].

From a gene-level perspective, different forms of Emery–Dreifuss muscular dystrophy (EDMD) can be distinguished according to the underlying mutations [3,4,5]. EDMD is a genetically heterogeneous disorder encompassing X-linked and autosomal forms, classically defined as X-linked EDMD (OMIM #310300) and autosomal dominant EDMD (OMIM #181350).

EDMD1 is the X-linked form caused by mutations in the *EMD* gene (OMIM #300384), which encodes the protein emerin, a component of the inner nuclear membrane, and represents the most frequently diagnosed form [5,6]. EDMD2 is the autosomal dominant form caused by mutations in the *LMNA* gene (OMIM #150330), encoding lamins A and C [5,7].

*LMNA* mutations are notable for their marked phenotypic pleiotropy, spanning a wide spectrum of laminopathies that include not only EDMD but also dilated cardiomyopathy with conduction system disease, often characterized by a disproportionate arrhythmic burden relative to left ventricular dysfunction [8,9,10,11].

Beyond *EMD* and *LMNA*, additional genes have been implicated in EDMD-like phenotypes, including *FHL1* (OMIM #300163) and genes encoding nesprins and other nuclear envelope-associated proteins (e.g., *SYNE1*, *SYNE2*, and *TMEM43*), further supporting a shared pathobiological framework centered on nuclear–cytoskeletal coupling and mechanotransduction [10,12]. EDMD4 and EDMD5 are caused by mutations in the *SYNE1* or *SYNE2* genes, respectively, encoding nesprin-1 and nesprin-2 [5,13].

Additional, less common EDMD subtypes have been described and further support the marked genetic heterogeneity of the disease. In particular, EDMD3 and EDMD6 have been linked to mutations in genes encoding nuclear envelope-associated or related proteins, although their clinical characterization remains limited. Moreover, EDMD7 has been associated with pathogenic variants in *TMEM43* (OMIM #612048), a gene classically implicated in arrhythmogenic cardiomyopathy [5,13,14]. These forms are rare and incompletely defined but reinforce the concept that EDMD represents part of a broader spectrum of nuclear envelope and nuclear–cytoskeletal disorders with overlapping cardiac phenotypes.

The disruption of these pathways leads to increased cellular susceptibility to mechanical stress, progressive myocyte damage, and electrical instability, which clinically manifest as early atrial involvement, conduction disease, and malignant ventricular arrhythmias [9,12,15].

From a clinical perspective, genetic testing plays a central role in the evaluation of suspected inherited cardiomyopathies and conduction disorders, particularly in the presence of early atrioventricular block, atrial standstill, or a positive family history. Current practice guidelines emphasize the importance of integrating genetic data into clinical decision-making, including family screening and risk stratification, especially for high-risk genotypes such as *LMNA*-related disease [11,16,17].

**Figure 1:**
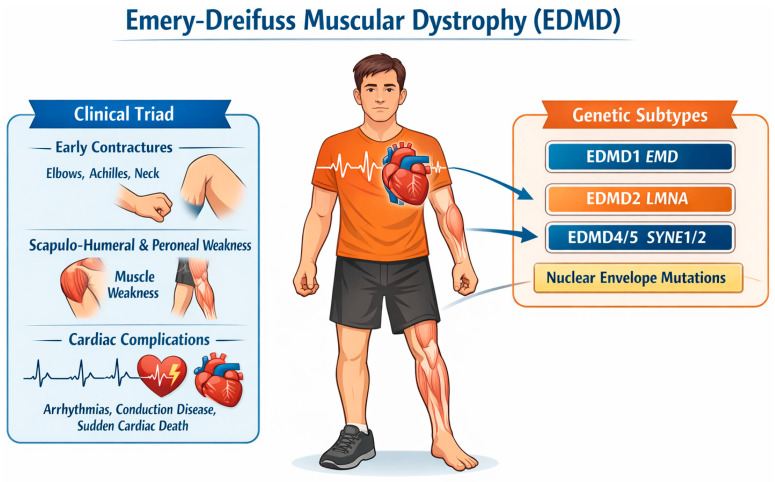
Classical clinical triad and major genetic determinants of EDMD. EDMD is classically defined by the association of early musculo-tendinous contractures, slowly progressive humeroperoneal muscle weakness, and cardiac involvement characterized by conduction disease, arrhythmias, and variable cardiomyopathy. The figure also highlights the principal genes implicated in EDMD, including EMD, *LMNA*, and other nuclear-envelope/LINC-complex-related genes, underscoring the genetic heterogeneity of the disorder and the close link between molecular substrate and clinical phenotype. Abbreviations: EDMD, Emery–Dreifuss muscular dystrophy; EMD, emerin gene; *LMNA*, lamin A/C gene; and LINC, linker of nucleoskeleton and cytoskeleton.

Figure 1 summarizes the classical clinical triad of EDMD together with the main genes involved in its pathogenesis.

A large-scale study conducted on cells from affected patients demonstrated that myogenic, metabolic, and fibrotic signaling pathways are strongly implicated. In particular, α-smooth muscle actin–positive myofibroblasts were found to be overrepresented, and the profibrotic miRNA-21 was upregulated. Consistently, correction of the mutation-carrying sequence was associated with the reduced expression of fibrogenic molecules [4].

From a clinical standpoint, disease progression is often characterized by the so-called “triad,” with early development of contractures, followed by fatigue and muscle atrophy, and ultimately the onset of cardiac abnormalities [6].

Notably, there is no strict correlation between muscular and cardiac manifestations. Cardiac involvement in EDMD may follow an independent course and can even precede neuromuscular symptoms [8]. In some cases, cardiomyopathy may present with syncope or sudden death, which may represent the first clinical manifestation in certain forms [18].

Current management remains largely symptomatic, aimed at mitigating orthopedic and cardiac complications, as no curative therapy is currently available [4]. The dissociation between cardiac and skeletal muscle involvement was further emphasized in a study by Ishikawa et al., which introduced the concept of “cardiac emerinopathy” to describe patients carrying *EMD* mutations who exhibit an isolated cardiac phenotype without clinically relevant neuromuscular manifestations [19].

The 2023 ESC Guidelines for the management of cardiomyopathies explicitly recognize laminopathies, including EDMD, as important causes of genetic cardiomyopathy and emphasize the need for an etiology-driven, multidisciplinary diagnostic approach rather than a purely morphological classification [16]. Given the central role of nuclear envelope dysfunction in EDMD, a detailed understanding of its genetic architecture and molecular mechanisms is essential to interpret the heterogeneous cardiac phenotype.

**Figure 2:**
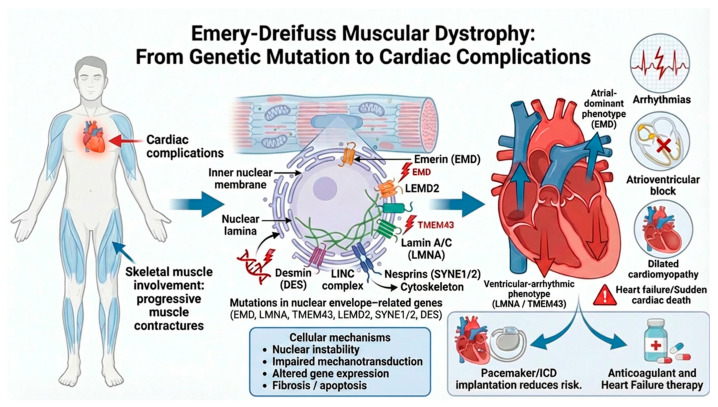
From genetic defect to cardiovascular complications in EDMD. Schematic representation of the pathophysiological continuum underlying cardiac involvement in EDMD. Pathogenic variants in nuclear envelope-related genes, classically including *EMD*, *LMNA*, *TMEM43*, *LEMD2*, *SYNE1/2*, and *DES*, lead to structural and functional disruption of the cardiomyocyte nucleus, with altered mechanotransduction, impaired nuclear stability, abnormal gene expression, and progressive fibro-fatty remodeling. These molecular and cellular abnormalities translate into a characteristic cardiac phenotype dominated by atrial myopathy, conduction system disease and arrhythmogenic cardiomyopathy. Clinically, this may manifest as supraventricular tachyarrhythmias, bradyarrhythmias, advanced atrioventricular block (AVB), ventricular arrhythmias, progressive ventricular dysfunction, heart failure, thromboembolic complications, and sudden cardiac death. The figure also summarizes the major therapeutic consequences of this disease trajectory, including pacemaker or implantable cardioverter–defibrillator implantation, anticoagulation in the setting of atrial disease and guideline-directed heart failure therapy when ventricular dysfunction develops. Abbreviations: EDMD, Emery–Dreifuss muscular dystrophy; *EMD*, emerin gene; ICD, implantable cardioverter–defibrillator; and *LMNA*, lamin A/C gene.

The pathophysiological link between the underlying genetic defect and the major cardiovascular manifestations of EDMD is schematically illustrated in Figure 2.

### 1.2. Molecular Genetics and Protein Network of Emery–Dreifuss Muscular Dystrophy

EDMD belongs to the expanding spectrum of nuclear envelope disorders, characterized by pathogenic variants affecting proteins involved in nuclear structure, mechanotransduction, and nucleo-cytoskeletal coupling [5,12]. The genetic architecture of EDMD is highly heterogeneous and extends beyond classical genes to a broader network of nuclear envelope and cytoskeletal components.

The most frequently implicated genes include *EMD* (OMIM #300384), encoding emerin, and *LMNA* (OMIM #150330), encoding lamin A/C [3,4,5,9,20]. Emerin is a LEM-domain protein localized at the inner nuclear membrane and involved in chromatin tethering and transcriptional regulation, whereas lamin A/C forms the nuclear lamina, providing structural stability and regulating genome organization and intracellular signaling pathways. In addition, lamin proteins play a central role in mechanosensing and cellular stress responses; their dysfunction leads to nuclear fragility, altered gene expression, and the activation of maladaptive signaling pathways, ultimately promoting apoptosis, fibrosis, and electrical instability in cardiomyocytes [4,15].

Beyond these classical forms, EDMD-related phenotypes also involve mutations in *SYNE1* and *SYNE2*, encoding nesprins, which are key components of the linker of nucleoskeleton and cytoskeleton (LINC) complex. This system physically connects the nuclear envelope to the cytoskeleton through SUN-domain proteins, enabling force transmission across the cell. Disruption of this network impairs mechanotransduction, nuclear positioning, and cellular signaling, particularly in mechanically stressed tissues such as cardiac muscle [9,12,15].

Additionally, less common EDMD subtypes further expand this genetic spectrum. EDMD4 and EDMD5 are associated with *SYNE1* and *SYNE2* variants, respectively, while EDMD7 has been linked to pathogenic variants in *TMEM43* (OMIM #612048), a gene more traditionally associated with arrhythmogenic cardiomyopathy. Other rare forms, including EDMD3 and EDMD6, have been described but remain incompletely characterized. Overall, these entities reinforce the concept of EDMD as a genetically and clinically heterogeneous continuum of nuclear envelope disorders [5].

Importantly, the genetic spectrum of EDMD overlaps substantially with primary cardiomyopathies. Pathogenic variants in *LMNA* are a well-established cause of dilated cardiomyopathy with prominent arrhythmic features, often disproportionate to left ventricular dysfunction [10,11,21]. In parallel, *EMD* mutations may lead to isolated cardiac phenotypes, including the so-called “cardiac emerinopathy” [15,19]. Similarly, *TMEM43* variants, classically associated with arrhythmogenic right ventricular cardiomyopathy type 5, may present with overlapping neuromuscular and cardiac features, supporting a continuum between neuromuscular disease and primary cardiomyopathy [7,13,14,21]. Experimental evidence further supports this concept, as the altered expression of *TMEM43* results in abnormal cardiac structure and impaired ventricular function, highlighting its role in nuclear envelope integrity and cardiac homeostasis [7,14].

Mutations in *LEMD2*, another LEM-domain protein of the inner nuclear membrane and structurally related to emerin, have also been linked to arrhythmogenic cardiomyopathy. These findings underscore shared molecular mechanisms across nuclear envelope disorders, including nuclear instability, chromatin disorganization, and impaired nuclear signaling [9,22].

Furthermore, the nuclear envelope is functionally coupled to the cytoskeleton through intermediate filaments, particularly desmin, encoded by *DES* (OMIM #125660). Desmin forms a structural network linking the sarcomere to the nucleus, and its disruption results in cardiomyopathy characterized by conduction disease and arrhythmias [10,23,24]. Notably, both lamin A/C and desmin belong to the intermediate filament protein family, providing a mechanistic bridge between nuclear and cytoplasmic structural defects and reinforcing the concept of integrated cellular architecture failure in EDMD and related disorders [10,15,23].

Collectively, these observations support a unifying model in which EDMD and related conditions can be conceptualized as a spectrum of “nuclear–cytoskeletal cardiomyopathies”, where the disruption of nuclear envelope integrity, LINC complex function, and intermediate filament networks converges toward a shared phenotype characterized by atrial myopathy, conduction disease, arrhythmias, and progressive cardiomyopathy [9,12,15].

Given the central role of nuclear envelope dysfunction in EDMD, a detailed understanding of its genetic architecture and molecular mechanisms is essential to interpret the marked heterogeneity of the cardiac phenotype and to support genotype-oriented risk stratification and management [11,16,17].

## 2. Materials and Methods

A targeted literature search was conducted in PubMed, Embase, and Web of Science to identify studies addressing cardiovascular involvement in EDMD. The search included original studies, case series, registries, guideline documents and reviews published up to January 2026. Search terms included combinations of the following: “Emery–Dreifuss muscular dystrophy”, “EDMD”, “EMD”, “LMNA”, “laminopathy”, “cardiac involvement”, “arrhythmias”, “atrial fibrillation”, “ventricular tachycardia”, “conduction disease”, “atrioventricular block”, “pacemaker”, “implantable cardioverter–defibrillator”, “cardiomyopathy”, “heart failure”, and “sudden cardiac death”.

Studies were selected based on their relevance to cardiovascular manifestations of EDMD, including genotype–phenotype correlations, arrhythmic risk, conduction system disease, cardiomyopathy progression, device-based and pharmacological management strategies. Articles were limited to those published in English. Given the narrative nature of the review, formal inclusion/exclusion criteria and quantitative synthesis were not applied; instead, evidence was critically appraised and integrated with emphasis on clinical relevance and methodological robustness.

Rare and under-recognized clinical presentations were also considered to provide a comprehensive and practice-oriented perspective.

This review does not present new or unpublished data. The manuscript was prepared in accordance with the recommendations of the International Committee of Medical Journal Editors for the conduct, reporting, editing, and publication of scholarly work in medical journals.

## 3. Epidemiology, Diagnostic Criteria, and Neuromuscular Involvement

EDMD is a rare disorder whose real prevalence remains uncertain, with estimates varying widely across sources and subtypes, ranging from approximately 1:400,000 to 1.3–2 per 100,000 (≈1:50,000–1:77,000). In the epidemiological literature, prevalence has been estimated at approximately 0.39 per 100,000 (≈1:256,000) up to 1:250,000 births, while X-linked EDMD has been reported at approximately 0.13 per 100,000 in a regional population study and more commonly at approximately 1:100,000 male births [6,25,26].

Despite its classical description, the epidemiology remains imprecise, largely due to marked genetic heterogeneity, incomplete penetrance, and frequent under-recognition, particularly in patients presenting with an apparently isolated cardiac phenotype [16,17].

### 3.1. Age and Sex Distribution

Age at presentation and sex distribution are strongly influenced by the underlying gene and mode of inheritance. In X-linked EDMD (EDMD1) due to pathogenic variants, the full clinical phenotype is observed almost exclusively in males, whereas heterozygous females typically exhibit a milder and later-onset presentation [27,28].

In a recent large multicentre cohort, males with EDMD1 had a mean age of 33.4 ± 13.3 years at clinical evaluation and exhibited a substantial risk of malignant ventricular arrhythmias and progression to end-stage heart failure. In contrast, female carriers were older (43.3 ± 16.8 years) and, although approximately 40–45% developed cardiac involvement during follow-up, this typically occurred later in life (median age ~58.6 years) and was associated with a markedly lower incidence of life-threatening ventricular arrhythmias [28]. Female carriers are often asymptomatic but may develop cardiac symptoms after the age of 50, particularly conduction abnormalities [29].

These data have direct clinical implications, supporting lifelong cardiac surveillance even in apparently asymptomatic female carriers. In autosomal dominant EDMD (EDMD2) associated with *LMNA* variants, both sexes are affected, and clinical expression is considerably more heterogeneous [9,30].

In these patients, disease onset frequently occurs in adulthood and may be dominated by a cardiac-first phenotype, characterized by early conduction system disease, atrial arrhythmias, or dilated cardiomyopathy, while skeletal muscle involvement may be subtle or clinically unapparent in the early stages [9,16].

This presentation is consistent with the 2023 ESC Guidelines for the management of cardiomyopathies concept that early arrhythmias and conduction disturbances may represent sentinel manifestations of genetic or syndromic cardiomyopathies [16].

### 3.2. Neuromuscular Involvement: The Diagnostic Core of EDMD

Despite increasing attention to the cardiac phenotype, neuromuscular involvement remains the most characteristic and diagnostically specific feature of EDMD, particularly in classical forms. Early and selective musculo-tendinous contractures are often the first clinical manifestation and may appear in childhood or adolescence [6,27].

Typical sites include the elbows (loss of extension), Achilles tendons (limited dorsiflexion or equinus deformity), and cervical and paraspinal muscles, leading to progressive spinal rigidity (“rigid spine”). These contractures frequently precede overt muscle weakness and represent one of the most discriminating clinical features of EDMD compared with other muscular dystrophies [6,31].

The associated myopathy is slowly progressive and displays a highly characteristic humeroperoneal distribution, with the involvement of the scapulohumeral muscles in the upper limbs (often with scapular winging) and distal peroneal compartments in the lower limbs, resulting in foot drop and a steppage gait [6,27]. Progression is typically slow, and muscle strength may remain relatively preserved for years, a feature that may delay diagnosis unless contractures are actively sought [30].

Spinal rigidity constitutes an additional hallmark. Progressive limitation of cervical and thoracic mobility may be disproportionate to the degree of muscle weakness and contributes significantly to functional impairment. Together, the triad of early contractures, humeroperoneal weakness, and rigid spine constitutes a powerful pattern-recognition framework for clinical diagnosis [27,30].

From a laboratory and electrophysiological standpoint, serum creatine kinase levels are often normal or only mildly elevated and therefore lack exclusionary value. Electromyography typically demonstrates non-specific myopathic changes. Muscle biopsy, historically used in the diagnostic work-up, is now reserved for selected cases, as genetic testing has become the diagnostic gold standard [16,27].

### 3.3. Peripheral Neuropathy: Accessory Feature and Overlap Phenotypes

In contrast to the prominent myopathic features, peripheral neuropathy is not considered a cardinal feature of classical EDMD [27]. Nevertheless, a limited number of reports, mainly involving *LMNA*-related disease, have described patients with combined myopathic and neurogenic features on nerve conduction studies and electromyography, suggesting a myopathy–neuropathy overlap phenotype [32,33].

Experimental and pathological data indicate that nuclear envelope dysfunction may, in rare cases, affect peripheral nerves or Schwann cells [33]. Clinically, the presence of mild peripheral neuropathy does not exclude EDMD when typical contractures and humeroperoneal myopathy are present. Conversely, a phenotype dominated by sensory–motor neuropathy in the absence of early contractures should prompt consideration of alternative diagnoses [27].

### 3.4. Integrated Diagnostic Criteria

Although no universally codified major/minor criteria exist, the contemporary diagnosis of EDMD relies on an integrated clinical–instrumental–genetic approach, consistent with the 2023 ESC Guidelines for the management of cardiomyopathies framework [16,34]:

- Typical neuromuscular phenotype: Early selective contractures plus humeroperoneal myopathy and/or rigid spine.
- Compatible cardiac phenotype: Conduction system disease, early atrial arrhythmias, ventricular arrhythmias, and/or heart failure.
- Genetic confirmation: Identification of a pathogenic or likely pathogenic variant in *EMD*, *LMNA*, or related nuclear envelope genes, followed by genetic counseling and cascade family screening.

This approach enables accurate diagnosis, prognostic stratification, and early identification of at-risk relatives. An integrated clinical–genetic diagnostic framework for EDMD is summarized in Table 1, highlighting how the combination of early contractures, humeroperoneal myopathy, cardiac involvement, and molecular confirmation supports timely diagnosis and family-based risk stratification.

Genotype–phenotype correlations in EDMD are summarized in Table 2, highlighting the clinically relevant distinction between the typically atrial-dominant course of *EMD*-related disease and the more malignant ventricular–arrhythmic profile usually observed in *LMNA*-related phenotypes.

## 4. Cardiac Involvement

### 4.1. Overview of Cardiac Complications in Emery–Dreifuss

Cardiac involvement in EDMD typically becomes clinically apparent from late adolescence to early adulthood, although subclinical abnormalities may precede overt manifestations [6,27,28]. Initial symptoms may include palpitations, presyncope, syncope, or reduced exercise tolerance, reflecting early electrical instability or chronotropic incompetence [2,6]. In some patients, sudden cardiac death may be the first manifestation, highlighting the malignant arrhythmic potential of the disease even in the absence of advanced structural remodeling [28,35].

Conduction system disease is a hallmark of EDMD and may affect multiple levels of the cardiac conduction axis [8,27]. The reported abnormalities include sinus bradycardia, sinoatrial block, atrial standstill, atrioventricular (AV) conduction delay, bundle branch block, and progression to complete AVB, frequently requiring permanent pacemaker implantation [8,19,36].

However, in selected patients with additional markers of arrhythmic risk, an implantable cardioverter–defibrillator (ICD) may be more appropriate than pacing alone [28,37]. Both atrial and ventricular arrhythmias are common during the disease course [38]. Atrial fibrillation and atrial flutter may occur at a young age and contribute substantially to thromboembolic risk, particularly in the setting of atrial mechanical dysfunction [19,39].

Ventricular ectopy and ventricular tachyarrhythmias also occur and are major determinants of prognosis [28,38]. With disease progression, structural cardiomyopathy and heart failure may emerge, most commonly with a dilated phenotype, while hypertrophic forms have been reported less frequently [28,40]. Importantly, sudden death may occur despite prior pacemaker implantation, indicating that pacing does not abolish arrhythmic risk [28,35,36]. In advanced cases, progression to end-stage heart failure may ultimately require heart transplantation as a definitive therapeutic option [28,41].

**Figure 3:**
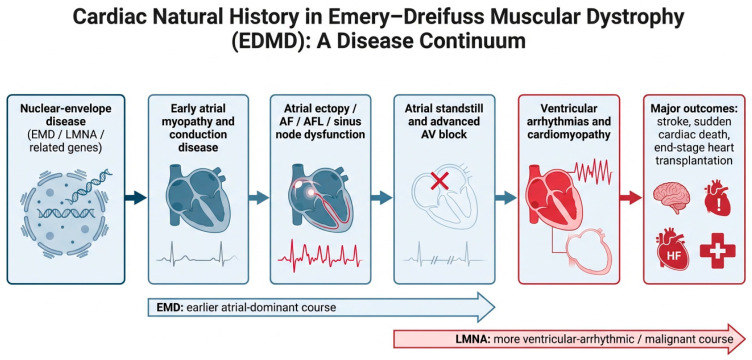
Natural history of cardiac involvement in EDMD. EDMD is characterized by a progressive cardio-neuromuscular phenotype in which early atrial disease and conduction abnormalities often precede overt ventricular dysfunction. The disease trajectory may evolve from atrial ectopy, atrial fibrillation/flutter, and sinus node dysfunction toward atrial standstill, advanced AVB, ventricular arrhythmias, cardiomyopathy, and major adverse outcomes including stroke, sudden cardiac death, end-stage heart failure, and heart transplantation. Although overlap is common, *EMD*-related disease more often shows an early atrial-dominant course, whereas *LMNA*-related disease is generally associated with a more malignant ventricular–arrhythmic phenotype. Abbreviations: AF, atrial fibrillation; AFL, atrial flutter; AVB, atrioventricular block; EDMD, Emery–Dreifuss muscular dystrophy; *EMD*, emerin gene; HF, heart failure; *LMNA*, lamin A/C gene.

The progressive cardiac trajectory of EDMD is illustrated in Figure 3, highlighting the transition from early atrial myopathy and conduction disease to atrial standstill, ventricular arrhythmias, cardiomyopathy, and major clinical outcomes.

The broad spectrum of cardiac involvement in EDMD is outlined in Table 3, underscoring that atrial disease, conduction abnormalities, ventricular arrhythmias, and progressive cardiomyopathy represent interconnected components of a single evolving cardio-neuromuscular phenotype.

### 4.2. Cardiac Imaging: Echocardiography and Cardiac Magnetic Resonance

Cardiac imaging, particularly echocardiography and cardiac magnetic resonance (CMR), plays a central role within a structured management strategy [2]. Transthoracic echocardiography commonly demonstrates a phenotype in which atrial remodeling may predominate over ventricular dilatation, with variable degrees of left ventricular (LV) systolic impairment and occasional diastolic dysfunction.

In a comparative echocardiographic study including both major genetic subgroups (*EDMD1*/*EMD* and *EDMD2*/*LMNA*), chamber enlargement (particularly atrial) was frequent, and a substantial subset had LVEF below the normal range, supporting the concept that structural and functional abnormalities may be present even in clinically “mild” skeletal phenotypes. Earlier echocardiographic series likewise documented that LV function can be subtly reduced compared with controls and that a minority of patients may fulfill criteria for dilated cardiomyopathy, reinforcing the need for systematic baseline and longitudinal echocardiographic surveillance [40,42].

A case–control echocardiographic study evaluated LV morphology and function in 27 men with confirmed EDMD (age 16–40 years; mean ~26 years), including 23 X-linked and 4 autosomal dominant cases, compared with 16 age-matched healthy male controls (mean ~25 years). The LV end-diastolic diameter was similar between groups, although three X-linked patients showed LV dilatation. Despite comparable wall thickness, EDMD patients exhibited evidence of LV remodeling, with higher relative wall thickness and increased LV mass index. LV systolic function was significantly reduced in EDMD, with a lower mean ejection fraction and 6/27 (22.2%) showing LVEF <50%; the most severe impairment (EF 19%, 32%, and 34%) occurred in X-linked disease, and overt heart failure was documented in one patient.

A Doppler assessment (in those in sinus rhythm) indicated impaired relaxation, with prolonged isovolumetric relaxation time, higher A-wave velocity, and reduced E/A ratio; applying age-specific criteria, slow early filling was present in four patients and a restrictive pattern in two with advanced systolic dysfunction. Overall, subclinical LV dysfunction was frequent even in young adults. A substantial proportion of patients, often minimally symptomatic, exhibit LV dysfunction, supporting the need for ongoing cardiology and echocardiographic surveillance [42].

Beyond standard parameters, deformation-based assessment suggests that myocardial dysfunction may be detectable at an early, subclinical stage. In autosomal dominant EDMD (*LMNA*-related), tissue Doppler and CMR-based strain analyses have identified abnormal LV functional indices despite preserved conventional chamber dimensions and the absence of late gadolinium enhancement, implying that functional impairment may precede overt scar formation [43].

In broader *LMNA* cardiomyopathy cohorts (highly relevant to EDMD2 and overlapping laminopathy phenotypes), speckle tracking-derived mechanical dispersion has been proposed as a marker associated with ventricular–arrhythmic risk beyond LVEF, supporting the rationale for incorporating strain mechanics into integrated phenotyping and follow-up when available [44].

CMR data in EDMD remain limited but are increasingly used to define an early myocardial phenotype beyond conventional echocardiography. In the single-center cohort described by Ditaranto et al., clinical trajectories differed according to neuromuscular onset, supporting the concept that *LMNA*-related disease spans heterogeneous cardiac phenotypes and progression patterns, with imaging playing a role in longitudinal characterization [45].

CMR is increasingly emphasized as the reference technique in muscular dystrophies to detect subclinical myocardial involvement and to support risk stratification, particularly through tissue characterization (e.g., LGE and mapping) and functional assessment [46]. Extending EDMD relevance to *EMD* variants, Bulmer et al. reported a large family with X-linked isolated dilated cardiomyopathy due to an *EMD* missense variant, in which CMR demonstrated late gadolinium enhancement with mixed ischaemic and non-ischaemic patterns, alongside conduction disease and ventricular arrhythmias, highlighting that a fibrotic/structural substrate may be present even when skeletal muscle involvement is minimal [47].

In EDMD2, feature-tracking/strain analyses have reported reduced inferior wall contractility compared with controls, suggesting that regional dysfunction may precede overt clinical cardiac manifestations; the absence of LGE argues against scar-driven impairment, unlike Duchenne muscular dystrophy, where LGE is common [43,48]. In *LMNA* mutation carriers, CMR more consistently identifies fibrosis: in a small cohort of asymptomatic or mildly symptomatic individuals, 88% had non-coronary, predominantly mid-myocardial basal septal LGE, often linear and involving < 50% of the affected segment [49].

Even with preserved LV systolic function, *LMNA* carriers may show longer phantom-normalized T2, higher extracellular volume (ECV), greater LGE burden, and impaired radial strain, consistent with subclinical cardiomyopathy; increased LGE burden and worse myocardial mechanics independently predicted a higher risk of major adverse cardiovascular events (MACE) [48,50].

As summarized in Table 4, multimodality imaging in EDMD extends beyond the conventional assessment of chamber size and ejection fraction, enabling earlier detection of subclinical myocardial dysfunction and more refined phenotypic characterization.

### 4.3. Heart Failure and Heart Transplantation

Unlike young patients with X-linked muscular dystrophies such as Duchenne muscular dystrophy, only a minority of individuals with EDMD develop dilated cardiomyopathy and heart failure [51].

In a longitudinal study of 18 patients with EDMD, progression to severe heart failure occurred in only one individual with an autosomal dominant form and severe limb–girdle muscular dystrophy, characterized by markedly reduced LVEF (30%), atrial flutter, and AVB requiring pacemaker implantation. Owing to refractory heart failure, the patient was ultimately referred for heart transplantation [39].

A non-negligible proportion of patients already exhibited advanced heart failure symptoms (NYHA functional class ≥III–IV) at the time of diagnosis and tended to progress further during follow-up. In some cases, heart failure severity may advance to the point of requiring heart transplantation. Most deaths in individuals with EDMD are attributable to sudden cardiac death or advanced heart failure [38].

Reversible forms of right-sided heart failure have been reported in patients with EDMD, particularly in the setting of venous congestion due to AVB with a very low heart rate, with improvement observed after pacemaker therapy [39].

### 4.4. ECG Abnormalities, Conduction Alterations and Brady-Arrhythmias

Atrial fibrillation and AV conduction disorders represent the most frequently observed arrhythmias, whereas life-threatening ventricular arrhythmias and sudden cardiac death occur less often but remain major complications [48].

Clinically relevant conduction abnormalities are commonly present already at the first cardiological assessment and tend to progress over time toward more advanced degrees of conduction block during longitudinal follow-up [15]. The surface ECG may show baseline abnormalities reflecting progressive involvement of the cardiac conduction system and/or an associated cardiomyopathic substrate, even in the absence of symptomatic rhythm disturbances.

Typical findings include low-amplitude P waves, AV conduction delay (PR prolongation up to higher-degree AV block), and intraventricular conduction disease with QRS widening (bundle branch block patterns or non-specific intraventricular conduction delay). Low QRS voltages and non-specific repolarization abnormalities (ST–T changes) may also be observed and should be interpreted within the broader clinical and imaging context, supporting the need for serial ECG surveillance as part of cardiac follow-up in EDMD/laminopathy phenotypes [28,30,52,53,54].

Sinus node disease and conduction abnormalities often precede the development of ventricular dysfunction [55]. Moreover, different degrees of AVB may occur [3,52]. However, in a systematic review by Valenti et al., sinus node disease or sinoatrial block was reported at baseline in only a small proportion of the included studies evaluating patients with cardiomyopathy [38].

It has been observed that patients carrying *LMNA* mutations often undergo pacemaker implantation for conduction disturbances, yet their risk of sudden cardiac death remains unchanged [56]. In EDMD, a true “conduction system disease” is present, manifesting as sinus node dysfunction (including atrial standstill) and AV conduction blocks, such as Mobitz type II AV block and complete AV block, which may ultimately require permanent pacemaker implantation [28].

The concomitant occurrence of bradyarrhythmias and supraventricular tachyarrhythmias is uncommon, particularly in younger patients; nonetheless, individuals with EDMD carry a substantial lifetime risk of developing both. Moreover, given the low escape rhythm rates that may occur in these patients, bradyarrhythmias have been reported as a potential mechanism of sudden cardiac death [39].

Regarding symptom burden, manifestations associated with complete AVB or sinoatrial block, such as syncope and/or fatigue, appear to be relatively well tolerated in patients with EDMD. This may reflect adaptation to chronically lower heart rates and/or reduced habitual physical activity compared with otherwise healthy young individuals [36].

### 4.5. Atrial Arrhythmias

Atrial involvement is a common component of the cardiac phenotype, and supraventricular tachyarrhythmias (SVTs) may precede the onset of overt cardiomyopathy, along a continuum ranging from atrial premature beats and atrial tachycardias to atrial fibrillation (AF) or flutter (AFL) and, particularly in more “atrial-dominant” phenotypes, progressing toward “atrial standstill” (atrial paralysis related to fibrosis).

Atrial standstill represents an extreme expression of atrial injury, characterized by the absence of P waves on ECG, atrial electrical silence with a junctional or ventricular escape rhythm, and lack of mechanical atrial contraction. Atrial ectopy should be interpreted as an early manifestation of a primary atrial myopathy related to nuclear envelope pathology, rather than merely an isolated electrical trigger [40].

From a pathophysiological perspective, the atrium is the target of progressive structural and electrical remodeling (substrate), upon which atrial premature beats act as markers and/or triggers of tachyarrhythmias. Atrial premature beats (APBs) and other supraventricular arrhythmias in EDMD arise from a distinctive substrate of primary atrial cardiomyopathy driven by mutations in *EMD* (emerin) or *LMNA* (lamin A/C). Nuclear lamina proteins play a critical role in nuclear architecture, mechanotransduction, and transcriptional regulation in cardiac cells; their dysfunction promotes interstitial fibrosis, fibro-fatty infiltration, and disruption of the atrial extracellular matrix, thereby generating abnormal conduction substrates with areas of slow conduction and re-entrant circuits that predispose to APBs, atrial tachycardias, and atrial fibrillation/flutter.

This atrial remodeling phenotype reduces local refractoriness, facilitates re-entry, and promotes abnormal automaticity, and may account for the high prevalence of supraventricular arrhythmias even in young patients, irrespective of the severity of ventricular dysfunction [39].

A systematic review reports that atrial tachyarrhythmias (AF, AFL, atrial tachycardia, and atrial standstill) are among the most frequently reported and quantifiable outcomes, consistent with the concept of progressive and clinically relevant atrial disease [38]. In a cohort of 45 patients with muscular dystrophies due to *EMD*/*LMNA* mutations and a long median follow-up, atrial arrhythmias (including atrial premature beats) were common and often emerged at a young age (in the second or third decade of life), particularly in emerinopathy, suggesting that atrial electrical instability can precede overt heart failure and ventricular remodeling.

Conversely, in laminopathy, ventricular arrhythmias appear more prevalent and tend to manifest earlier, underscoring distinct natural histories with an “early atrial-dominant” trajectory in *EMD* versus an “early ventricular-dominant” trajectory in *LMNA*. This suggests that atrial electrical instability may precede overt ventricular remodeling by years [40].

The overall reported prevalence of arrhythmias is approximately 89%, with atrial standstill in 31% and AF/AFL in 29% [40]. The incidence rate for AF/AFL/AT ranges from 6.1 to 13.9 events per 100 patient-years, whereas the incidence of atrial standstill ranges from zero to two events per 100 patient-years [38]. Approximately half of patients with *EMD* mutations develop atrial standstill, whereas this condition is not observed in the *LMNA* subgroup, suggesting a further difference in natural history between emerinopathy and laminopathy [40].

From a prognostic standpoint, the onset of atrial tachyarrhythmias (and, plausibly, an increasing burden of atrial ectopy) should be regarded as a marker of progression of atrial disease toward atrial paralysis and thromboembolic complications, and therefore as a signal to intensify surveillance and refine antithrombotic strategies [8,39].

Atrial standstill in EDMD is clinically pivotal because it often represents the end stage of a continuum of atrial tachyarrhythmias and bradyarrhythmias, with direct implications for pacing strategy, anticoagulation decisions, and ischaemic risk. Notably, reports that atrial standstill may emerge after phases of atrial fibrillation or flutter, followed by a progressively electrically “silent” atrium, support an evolution toward extensive atrial fibrosis, low-voltage substrate, and the loss of atrial capture, rather than a mere disorder of atrial automaticity [37,39,40].

Operational diagnosis of atrial ectopy and atrial tachycardias should be proactive and longitudinal, combining a baseline 12-lead ECG, 24 h Holter monitoring (preferably extended over multiple days), and, when available, pacemaker/ICD diagnostics, as continuous surveillance can capture nocturnal or intermittent atrial arrhythmias that may be missed during routine outpatient assessment [8,40,57].

Thromboembolic event rates in EDMD have been reported to be as high as 8.9 events per 100 patient-years, consistent with the substantial ischaemic burden observed in cohort studies and supporting a proactive (non-watchful waiting) prevention strategy [38]. Anticoagulation should be recommended when AF/AFL occurs in EDMD and should also be considered in atrial standstill, given the consistently reported high thromboembolic risk and its disproportionate prognostic impact relative to patients’ age. In the absence of EDMD-specific trials or dedicated guidelines, management is extrapolated from standard AF anticoagulation principles in structural cardiomyopathies and individualized to the disease context.

The 2022 HRS consensus statement on neuromuscular diseases (including EDMD) emphasizes systematic arrhythmia surveillance and structured thromboembolic risk assessment, explicitly noting that the combination of atrial arrhythmias/atrial standstill and stroke warrants a rigorous, guideline-consistent approach to anticoagulation when indicated [37].

In a long-term longitudinal study including X-linked and autosomal dominant EDMD, AF/AFL developed in 11/18 patients (61%), followed by atrial standstill in 5/11 (45%), while embolic stroke occurred in 4/11 (36%) of AF/AFL patients, often with disabling sequelae. This suggests a clinically relevant temporal sequence of “AF/AFL → atrial standstill → stroke” in a non-negligible proportion of cases. Accordingly, the most robust clinical rationale for anticoagulation in EDMD derives from these longitudinal data, where the authors conclude that antithromboembolic prophylaxis should be recommended in the presence of AF/AFL or atrial standstill [8,16,37].

In “cardiac emerinopathy” (the X-linked cardiac phenotype related to *EMD*, even in the absence of a full syndromic presentation), a trajectory of progressive atrial arrhythmias culminating in atrial standstill has been described, frequently in association with left ventricular non-compaction and a familial thromboembolic burden, reinforcing the concept of a structured cardiomyopathy/atriomyopathy with an intrinsic embolic risk [19].

As shown in a multicentre cohort of *EMD* variant carriers, even when the earliest clinical manifestations are predominantly atrial, early and structured surveillance remains essential, given the substantial risk of subsequent malignant ventricular arrhythmias and progression to end-stage heart failure [28].

There are no dedicated randomized trials assessing the efficacy of antiarrhythmic drugs for atrial arrhythmias; therefore, pharmacological management is extrapolated from general principles for atrial tachyarrhythmias, with particular attention to the underlying conduction substrate and the frequent coexistence of bradyarrhythmias that characterize these dystrophies. Medical therapy is based on rate and/or rhythm control using antiarrhythmic agents selected according to ventricular function, conduction disturbances, and proarrhythmic risk, bearing in mind that management is often device-integrated in the context of a brady–tachy syndrome [16,37].

### 4.6. Ventricular Arrhythmias and Sudden Cardiac Death

Malignant ventricular arrhythmias are common in patients with EDMD, irrespective of left ventricular ejection fraction, and are associated with an increased risk of sudden cardiac death [55].

The risk of ventricular arrhythmias (VAs) in this condition has long been recognized [31,35]. The incidence of VAs appears to be higher in the presence of significant left ventricular systolic dysfunction, similarly to other forms of heart disease [58]. However, the true risk of progression to sudden death in EDMD patients receiving prophylactic device implantation despite preserved left ventricular function remains uncertain [39].

In EDMD (both *EMD*/emerin and *LMNA*/lamin A/C phenotypes), ventricular arrhythmias may occur as part of a broader “conduction–cardiomyopathy” continuum and can emerge even before advanced LV dysfunction becomes evident. From an ECG standpoint, the most reproducible correlations are not linked to a specific lead “location,” but rather to the burden of conduction disease and its progression over time: marked PR prolongation/AV conduction delay and QRS widening (bundle branch block or non-specific intraventricular conduction delay), particularly when these parameters worsen on serial ECGs, are associated with a more arrhythmogenic phase in cardiac laminopathies [28,37,59].

Cardiac magnetic resonance adds anatomical specificity: septal late gadolinium enhancement (LGE), a typical pattern in *LMNA*-related disease, has been associated with a higher incidence of major ventricular arrhythmias and helps refine risk stratification beyond surface ECG alone [60].

In EDMD, *LMNA*-related cardiomyopathy appears to follow a particularly malignant course compared with other dilated cardiomyopathy aetiologies, with malignant ventricular arrhythmias and progressive end-stage, medically refractory heart failure more frequently observed in *LMNA* mutation carriers [15,21,61]. A similar trend has also been reported in *LMNA* mutation carriers with a left ventricular ejection fraction > 35% [21].

Patients with *LMNA* mutations have a high risk of premature sudden cardiac death, likely driven by ventricular tachyarrhythmias; this risk does not parallel the onset of heart failure or bradyarrhythmias and, accordingly, is not prevented by pacemaker implantation alone [56,62]. In a multicentre study including 59 patients with EDMD type 1 (*EMD* variant carriers), a 50-year-old man with a permanent pacemaker died suddenly. Post-mortem device interrogation documented rapid sustained ventricular tachycardia coinciding with collapse, and an echocardiogram performed two weeks earlier showed only mildly reduced LVEF (43%), supporting the concept that malignant ventricular arrhythmias may occur despite the absence of severe LV systolic dysfunction [28].

In *LMNA* mutation carriers, the use of a recently validated risk score has been proposed. This clinically useful tool was developed from a multicentre study involving 839 patients recruited from two registries (660 from the French Nationwide Registry and 179 from international cohorts). The aim of the study was to validate a model to estimate the 5-year absolute risk of developing life-threatening ventricular tachyarrhythmias (LTVTA) in patients with dilated cardiomyopathy caused by *LMNA* mutations, typically with adult-onset disease.

The study population included 55 patients with EDMD from the French registry and 6 additional EDMD patients from other registries (including London, the United States, Bern, and Melbourne). LTVTA was defined as the occurrence of the following:

- Sudden cardiac death;
- Appropriate ICD shock for termination of ventricular tachyarrhythmias;
- Ventricular tachyarrhythmias associated with haemodynamic instability and clinical manifestations.

The exclusion criteria included a history of tachyarrhythmias prior to baseline, age < 16 years, onset of neuromuscular or systemic disease before the age of 16 years, cardiomyopathies associated with other genetic mutations, lack of clinical data, and the first cardiological evaluation being performed before January 2000.

The risk score is based on the presence of several adverse factors associated with an increased risk of malignant ventricular arrhythmias in laminopathies:

- Male sex;
- Baseline left ventricular ejection fraction < 45%;
- Non-missense genetic variants;
- Non-sustained ventricular tachycardia;
- AVB.

The score can be readily calculated in these patients, including those with EDMD due to *LMNA* mutations and adult-onset dilated cardiomyopathy, using the dedicated online calculator (https://lmna-risk-vta.fr/, accessed on 20 April 2026). Risk estimation is crucial for identifying patients who may benefit from ICD implantation, helping to avoid both the under- and overestimation of risk [11].

### 4.7. Cardiac Implantable Electronic Device and Ablation Therapy

In EDMD, normal myocardium is gradually replaced by fibrous and adipose tissue, a process that typically begins in the atria and subsequently involves the AV node and ventricles. This electro-anatomical substrate underlies the frequent occurrence of bradyarrhythmias and tachyarrhythmias, which represent a predominant feature of the disease.

Conduction abnormalities, such as sick sinus syndrome (often associated with atrial standstill) and various degrees of AVB, are common and may be associated with the development of cardiomyopathy. According to ESC guidelines, permanent pacing is indicated (Class I recommendation) in cases of second- or third-degree AVB or HV interval ≥ 70 ms, regardless of symptoms, and may be considered (Class IIb) in patients with PR interval ≥240 ms or QRS duration ≥120 ms [63].

In a case series by Steckiewicz et al. including 21 patients with *EMD* mutations followed for a mean of 11 ± 8 years after pacemaker (PM) implantation, the pacing mode was frequently changed from DDD to VVI during follow-up due to atrial electrical silence. This phenomenon was associated with marked atrial spike-to-P-wave delay, the loss of atrial capture, and a high incidence of atrial fibrillation/flutter [36].

Whenever pacing is indicated in this disease, an implantable cardioverter–defibrillator (ICD) should be considered according to guideline recommendations. In the report by Golzio et al., ventricular lead extraction was required only 18 months after PM implantation. The patient subsequently underwent device upgrade to an ICD for ventricular tachycardia, and nine months later multiple appropriate ICD therapies successfully prevented sudden cardiac death [64].

Sudden cardiac death is not uncommon in EDMD, underscoring the need for appropriate risk stratification [65]. As indicated in the 2022 ESC guidelines for the management of ventricular arrhythmias, risk assessment should not be limited to patients with heart failure and left ventricular ejection fraction < 35%, but should also be considered in those who do not meet conventional criteria when additional risk factors are present. These include age, a family history of sudden cardiac death, ECG conduction abnormalities (e.g., PR prolongation and left bundle branch block), atrial arrhythmias, non-sustained ventricular tachycardia, left ventricular dysfunction (LVEF < 45%), structural abnormalities on CMR, and *LMNA* mutations [66].

Cardiac resynchronization therapy may also have a role in the management of patients with EDMD, given the high incidence of heart failure in dilated cardiomyopathy, particularly among *LMNA* mutation carriers, and the frequent presence of intraventricular conduction disturbances. In 1999, Walker et al. reported the first anecdotal case of biventricular ICD implantation [67]. To date, however, no systematic studies have evaluated outcomes or responsiveness to resynchronization therapy in EDMD. Similarly, no data are available on emerging selective pacing techniques, such as left bundle branch area pacing or His bundle pacing, in this setting.

The prevalence of atrial arrhythmias ranges from 12.5% to 63% in *LMNA* cohorts and from 10% to 73.3% in *EMD* cohorts, while malignant ventricular arrhythmias are also frequent, particularly in *LMNA*-mutated patients. In a systematic review, ICD implantation resulted in device activation in 24% to 52.4% of carriers [38].

Catheter ablation therapy may be challenging due to regional and transmural repolarization heterogeneity and electrical instability related to diffuse fibrosis. Only three reported cases of ablation in EDMD patients are available. Blagova described a case of atrial flutter ablation that was initially successful but recurred within four months, complicated by worsening LV systolic function requiring heart transplantation [41]. Carvalho reported a case involving multiple ablation procedures for atrial and ventricular tachyarrhythmias [68]. In 2020, Butt described the only successful case of atrial flutter ablation via cavotricuspid isthmus ablation, with arrhythmia-free survival at 35 months of follow-up [69].

These anecdotal cases highlight the complexity of the arrhythmogenic substrate in EDMD. Moreover, data on ventricular arrhythmia ablation in EDMD are scarce, possibly reflecting limited accessibility or the perceived futility of the procedure. Valverde Soria described a patient with EDMD-related dilated cardiomyopathy (*EMD*-related phenotype) and an ICD who developed recurrent ventricular fibrillation episodes triggered by short-coupled monomorphic ventricular ectopy, consistent with a Purkinje-related focus within an intraseptal substrate. A mechanism-oriented strategy combining substrate ablation with ICD-guided pace mapping successfully eliminated the trigger and controlled recurrences, supporting catheter ablation of Purkinje triggers as a potential adjunctive option in selected cases [70].

The review by Zeppenfeld provides a practical, mechanism-based framework for ventricular tachycardia ablation in non-ischaemic cardiomyopathy, although it does not specifically address EDMD. Nevertheless, these concepts may reasonably be extrapolated to EDMD patients who develop sustained monomorphic ventricular tachycardia or recurrent ICD shocks. Non-ischaemic ventricular tachycardia is often driven by scar-related re-entry with heterogeneous, sometimes intramural or epicardial substrate distribution, which may limit ablation efficacy and contribute to recurrence. Recognition of His–Purkinje system-dependent ventricular tachycardias (including bundle branch re-entry) is particularly important, as these may represent high-yield ablation targets in selected non-ischaemic phenotypes [71].

Finally, a pragmatic management framework for the principal cardiac scenarios encountered in EDMD is provided in Table 5, emphasizing the need for proactive surveillance, early recognition of thromboembolic risk, and individualized device-based decision-making.

## 5. Current and Future Directions

The 2023 ESC cardiomyopathy guidelines support earlier consideration of primary prevention ICD implantation in genotype-positive dilated cardiomyopathy or non-dilated left ventricular cardiomyopathy (NDLVC), including high-risk genotypes with additional markers (e.g., syncope or LGE), even in the absence of overt LV dysfunction. An ICD may also be considered in genotype-positive patients with LVEF >35% even without additional risk factors (Class IIb) [16].

In EDMD, these principles are biologically plausible but remain insufficiently validated; future studies should therefore prospectively assess genotype–substrate–event relationships, define actionable risk thresholds, and explore precision therapeutics (including pathway-targeted strategies and emerging gene- and RNA-based approaches) within multidisciplinary cardio-neuromuscular programs.

Future research in EDMD should adopt a genotype-driven approach, integrating systematic panel testing (*EMD*, *LMNA*, *SYNE1/2*, and related nuclear envelope/LINC genes) with deep phenotyping and cascade screening to refine prognostication and enable earlier, mechanism-based interventions. Patient-derived cellular models now provide actionable biological insights: across EDMD1/2/5, dermal fibroblasts exhibit a convergent profibrotic program characterized by increased TGF-β signaling and miR-21 upregulation, alongside the over-representation of α-SMA-positive myofibroblasts. Importantly, CRISPR/Cas-based correction (or allele-specific disruption) rescues nuclear envelope abnormalities and largely normalizes the disease-associated miRNA signature, supporting proof-of-principle strategies for gene correction and biomarker discovery using isogenic lines and iPSC-based platforms [4].

Future directions in EDMD should move from a predominantly phenotype-based approach toward a genotype-driven framework, integrating systematic molecular diagnosis (*EMD*/*LMNA*-first, with expanded cardiomyopathy/arrhythmia panels where appropriate), longitudinal variant reclassification, and cascade testing to better define penetrance, sex-specific expressivity, and age-related risk. This will require EDMD-specific longitudinal cohorts combining genomics with deep phenotyping (including ECG/Holter burden, atrial myopathy metrics, CMR tissue characterization, circulating biomarkers, and digital surveillance), as well as the development and validation of EDMD-specific risk models for malignant ventricular arrhythmias and end-stage heart failure.

**Figure 4:**
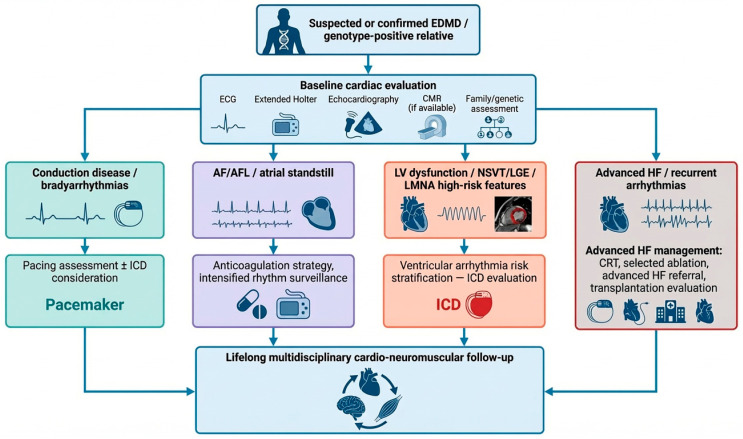
Pragmatic workflow for cardiac surveillance and clinical decision-making in EDMD. Patients with suspected or confirmed EDMD, as well as genotype-positive relatives, require structured baseline cardiac assessment based on electrocardiography, prolonged rhythm monitoring, echocardiography, and cardiac magnetic resonance when available. Findings such as conduction disease, atrial arrhythmias or atrial standstill, ventricular–arrhythmic markers, and progressive ventricular dysfunction should guide individualized decisions regarding pacing, anticoagulation, implantable cardioverter–defibrillator therapy, catheter ablation in selected cases, and referral for advanced heart failure therapies. Lifelong multidisciplinary follow-up is essential across all disease stages. Abbreviations: AF, atrial fibrillation; AFL, atrial flutter; CMR, cardiac magnetic resonance; CRT, cardiac resynchronization therapy; ECG, electrocardiogram/electrocardiography; EDMD, Emery–Dreifuss muscular dystrophy; HF, heart failure; ICD, implantable cardioverter–defibrillator; LGE = late gadolinium enhancement; *LMNA*, lamin A/C gene; LV, left ventricle/left ventricular; and NSVT, non-sustained ventricular tachycardia.

A pragmatic surveillance and management pathway for EDMD is proposed in Figure 4, integrating baseline phenotyping with subsequent decision-making regarding anticoagulation, pacing or defibrillator therapy, arrhythmia management, and referral for advanced heart failure therapies.

## 6. Conclusions

EDMD is a paradigmatic nuclear envelope disorder in which cardiac disease is not a late “complication” of skeletal myopathy but often an early and independent driver of morbidity and mortality. Across genotypes, the dominant clinical trajectory is characterized by a progressive atriomyopathy–conduction disease axis, with early atrial arrhythmias evolving toward atrial standstill and advanced bradyarrhythmias, variably accompanied by ventricular arrhythmias, cardiomyopathy, and heart failure.

Critically, malignant ventricular tachyarrhythmias and sudden cardiac death may occur out of proportion to left ventricular dysfunction and are not mitigated by pacemaker therapy alone, supporting earlier and more individualized consideration of ICD therapy, particularly in *LMNA*-related disease, within an etiology-driven framework aligned with contemporary ESC cardiomyopathy and ventricular arrhythmia guidelines.

From a clinical standpoint, EDMD mandates lifelong, structured cardiac surveillance integrating serial ECG and extended rhythm monitoring, device diagnostics when present, and multimodality imaging (echocardiography and, when available, CMR with tissue characterization) to capture the evolving substrate and refine risk stratification. Management should be explicitly proactive with regard to thromboembolic prevention, given the high burden of atrial disease and the recurrent sequence of AF/AFL progressing to atrial standstill and stroke.

While pharmacological and device-based strategies remain the cornerstone of care, evidence supporting catheter ablation in EDMD remains limited; however, selected cases suggest that mechanism-targeted approaches may be feasible in carefully selected patients.

Finally, the field is poised to transition from descriptive phenotyping toward genotype-driven precision care, leveraging deep longitudinal cohorts, EDMD-specific risk models, and translational pipelines (including isogenic systems and emerging gene- and RNA-based strategies) aimed at disease modification. Until such data become available, optimal outcomes will depend on early recognition, multidisciplinary cardio-neuromuscular management, and anticipatory prevention of stroke, heart failure progression, and sudden cardiac death.

## Figures and Tables

**Table 1:** Integrated diagnostic framework for EDMD: from neuromuscular phenotype to genetic confirmation and cardiac screening. The table summarizes the contemporary diagnostic approach to EDMD, integrating the characteristic neuromuscular phenotype with cardiac manifestations, family history, and molecular confirmation. This framework reflects the current shift from a purely descriptive syndrome-based diagnosis to an etiology-driven, multidisciplinary model aimed at early recognition, prognostic stratification, and cascade family screening. Abbreviations: CK, creatine kinase; CMR, cardiac magnetic resonance; ECG, electrocardiogram/electrocardiography; EDMD, Emery–Dreifuss muscular dystrophy; EMG, electromyography; *EMD*, emerin gene; *LMNA*, lamin A/C gene; LINC, linker of nucleoskeleton and cytoskeleton; *SYNE1*, spectrin repeat containing nuclear envelope protein 1 gene; and *SYNE2*, spectrin repeat containing nuclear envelope protein 2 gene.

Domain	Key Features	Clinical Relevance	Diagnostic Tools	Implications for Management
Neuromuscular phenotype	Early selective contractures involving elbows, Achilles tendons, and cervical/paraspinal muscles; humeroperoneal muscle weakness; rigid spine	Core phenotypic hallmark of classical EDMD and major clue for clinical suspicion	Neurological examination, musculoskeletal assessment, functional evaluation	Triggers targeted cardiological assessment and genetic testing
Skeletal muscle laboratory/electrophysiology	Serum CK often normal or only mildly elevated; EMG usually shows non-specific myopathic changes	Helpful as supportive but non-definitive findings; normal CK does not exclude EDMD	Serum CK, electromyography	Supports integrated work-up; should not delay molecular testing
Peripheral neuropathy/overlap phenotype	Occasional neurogenic features, mainly in selected *LMNA*-related cases; myopathy–neuropathy overlap may occur	Does not exclude EDMD when the typical contracture–myopathy pattern is present; helps refine phenotype	Nerve conduction studies, EMG, neurological re-evaluation	Broadens differential diagnosis and may justify extended genetic testing
Cardiac phenotype	Conduction disease, sinus node dysfunction, atrial fibrillation/flutter, atrial standstill, ventricular arrhythmias, cardiomyopathy, heart failure	Major determinant of morbidity and mortality; may precede overt neuromuscular manifestations	12-lead ECG, prolonged Holter monitoring, echocardiography, CMR when available	Enables early rhythm surveillance, anticoagulation assessment, and device-based risk stratification
Family history	Sudden cardiac death, pacemaker implantation, cardiomyopathy, muscular dystrophy, stroke, unexplained syncope	Strengthens suspicion of inherited cardio-neuromuscular disease	Pedigree analysis, family interview	Supports cascade screening and anticipatory evaluation of relatives
Genetic confirmation	Pathogenic/likely pathogenic variants in *EMD*, *LMNA*, *SYNE1*, *SYNE2*, or related nuclear-envelope/*LINC* complex genes	Confirms diagnosis, refines prognosis, and supports genotype-oriented follow-up	Targeted gene testing, cardiomyopathy/neuromuscular gene panels, variant interpretation	Enables counseling, longitudinal risk stratification, and family screening
Female carrier/genotype-positive relative evaluation	Female *EMD* carriers or asymptomatic relatives may show delayed or isolated cardiac involvement	Clinically relevant because cardiac disease may emerge late and independently of skeletal manifestations	ECG, Holter, echocardiography, genetic counseling	Supports lifelong cardiac surveillance even in apparently asymptomatic carriers
Integrated final diagnosis	Combined recognition of typical neuromuscular pattern, compatible cardiac phenotype, and molecular confirmation	Contemporary diagnosis is multidisciplinary and etiology-driven rather than purely descriptive	Joint neurology–cardiology–genetics assessment	Supports individualized monitoring and prevention of arrhythmic, thromboembolic, and heart failure complications

**Table 2:** Genotype–phenotype correlations in EDMD. The table summarizes the main clinical differences across EDMD-related genetic subgroups, emphasizing how *EMD*-associated disease is often characterized by an earlier atrial-dominant course, whereas *LMNA*-related disease typically carries a more malignant ventricular–arrhythmic and heart failure trajectory. Less common nuclear-envelope/LINC-complex phenotypes remain less well defined and require individualized interpretation. Abbreviations: AF, atrial fibrillation; AFL, atrial flutter; AV, atrioventricular; EDMD, Emery–Dreifuss muscular dystrophy; *EMD*, emerin gene; HF, heart failure; ICD, implantable cardioverter–defibrillator; *LMNA*, lamin A/C gene; LINC, linker of nucleoskeleton and cytoskeleton; NSVT, non-sustained ventricular tachycardia; VF, ventricular fibrillation; and VT, ventricular tachycardia.

Genotype/Disease Subgroup	Inheritance	Typical Extra-Cardiac Phenotype	Predominant Cardiac Phenotype	Characteristic Clinical Course	Main Management Implications
*EMD*-related EDMD (EDMD1/emerinopathy)	X-linked	Classical early contractures, humeroperoneal weakness, rigid spine; full phenotype mainly in males; female carriers often milder and later	Early atrial disease and conduction abnormalities; AF/AFL, atrial standstill, sinus node dysfunction, AV block; ventricular dysfunction may occur later	Often atrial-dominant in the early phase, with progression from atrial ectopy/tachyarrhythmias to atrial standstill and thromboembolic risk; malignant ventricular arrhythmias may still occur in selected patients	Lifelong rhythm surveillance; strong attention to atrial arrhythmias, atrial standstill, and thromboembolic prevention; pacing frequently required, but ICD may be needed in selected higher-risk cases
*LMNA*-related EDMD/laminopathy (EDMD2 and overlapping laminopathic cardiomyopathy phenotypes)	Usually autosomal dominant	Neuromuscular phenotype may be classical, subtle, delayed, or even overshadowed by cardiac manifestations; both sexes affected	Conduction disease, atrial arrhythmias, ventricular arrhythmias, dilated cardiomyopathy, progressive LV dysfunction, heart failure	Often more ventricular–arrhythmic and malignant, with higher risk of NSVT/VT/VF, sudden cardiac death, and end-stage heart failure; cardiac-first presentation is common	Early ICD-oriented risk stratification is crucial; CMR and *LMNA*-specific risk models may refine prognosis; surveillance must address both arrhythmias and HF progression
SYNE1/SYNE2-related EDMD (EDMD4/5 and related LINC-complex phenotypes)	Usually autosomal dominant or recessive depending on variant/context	Variable contractures and myopathy; phenotype often heterogeneous and less classically defined than *EMD*/LMNA forms	Cardiac phenotype less well characterized; may include conduction abnormalities, arrhythmias, and cardiomyopathic features	Natural history remains less clearly defined because of limited cohort data; penetrance and severity appear variable	Requires individualized follow-up within inherited cardio-neuromuscular programs; management usually extrapolated from broader EDMD/laminopathy principles
Female *EMD* carriers/genotype-positive relatives with limited neuromuscular phenotype	X-linked carrier state	Often absent, subtle, or delayed skeletal manifestations	Late-onset conduction disease, atrial arrhythmias, and other isolated cardiac manifestations may occur	Cardiac disease may emerge independently of overt muscular phenotype, often later in life	Cardiac surveillance should not be omitted on the basis of a mild skeletal phenotype or female sex
“Cardiac emerinopathy”/isolated or cardiac-predominant *EMD* phenotype	X-linked	Minimal or absent clinically relevant skeletal muscle involvement	Progressive atrial arrhythmias, atrial standstill, conduction disease, stroke risk; possible ventricular involvement/non-compaction in selected cases	Demonstrates that cardiac disease can dominate the phenotype and may be the presenting manifestation	Highlights the need to suspect EDMD-spectrum disease even in apparently isolated inherited arrhythmic/conduction phenotypes

**Table 3:** Spectrum of cardiac involvement in EDMD: major manifestations, clinical consequences, and monitoring implications. Cardiac disease in EDMD includes a broad continuum including conduction system disease, atrial myopathy with tachyarrhythmias and atrial standstill, ventricular arrhythmias, cardiomyopathy, heart failure, and thromboembolic complications. The table emphasizes the prognostic impact of each domain and the monitoring tools most relevant to longitudinal follow-up. Abbreviations: AF, atrial fibrillation; AFL, atrial flutter; AV, atrioventricular; CMR, cardiac magnetic resonance; EDMD, Emery–Dreifuss muscular dystrophy; ECG, electrocardiogram/electrocardiography; HF, heart failure; ICD, implantable cardioverter–defibrillator; LV, left ventricle/left ventricular; NSVT, non-sustained ventricular tachycardia; NYHA, New York Heart Association; VF, ventricular fibrillation; and VT, ventricular tachycardia.

Cardiac Domain	Typical Manifestations	Main Clinical Consequences	Prognostic Significance	Preferred Monitoring Tools
Conduction system disease/bradyarrhythmias	Sinus bradycardia, sinoatrial block, first-degree AV block, bundle branch block, Mobitz II AV block, complete AV block	Fatigue, presyncope, syncope, pacemaker requirement, low-output symptoms	Hallmark of EDMD; often progressive and may precede overt cardiomyopathy	Serial ECG, prolonged Holter monitoring, device interrogation
Atrial myopathy and atrial ectopy	Atrial premature beats, atrial tachycardia, electrical instability at a young age	Symptoms, progression to AF/AFL, marker of evolving atrial disease	Often an early manifestation, especially in *EMD*-related disease	ECG, extended rhythm monitoring, device diagnostics when available
Atrial fibrillation/atrial flutter	Paroxysmal or persistent AF/AFL, often occurring early in life	Palpitations, haemodynamic intolerance, thromboembolic risk, progression to atrial standstill	Major determinant of stroke risk and marker of advanced atrial remodeling	ECG, prolonged Holter, device diagnostics, echocardiographic atrial assessment
Atrial standstill	Absence of P waves, atrial electrical silence, lack of atrial contraction, loss of atrial capture	Severe bradycardia, ineffective atrial pacing, thromboembolism, stroke	End-stage expression of atrial disease with major pacing and anticoagulation implications	ECG, echocardiography, device interrogation
Ventricular arrhythmias	Ventricular ectopy, NSVT, sustained VT, VF	Sudden cardiac death, ICD therapies, recurrent hospitalizations	One of the main determinants of prognosis, particularly in *LMNA*-related disease	Holter monitoring, ICD diagnostics, CMR, risk score integration in *LMNA*
Structural cardiomyopathy	Atrial enlargement, LV systolic dysfunction, dilated phenotype, occasional regional dysfunction	Reduced exercise tolerance, progressive ventricular remodeling, arrhythmic substrate	Variable across genotypes but strongly linked to HF progression and arrhythmic burden	Echocardiography, CMR, serial imaging follow-up
Heart failure	LV dysfunction, NYHA III–IV symptoms, progressive congestion, end-stage disease	Hospitalization, reduced survival, transplantation in selected cases	Major cause of mortality together with sudden cardiac death	Clinical follow-up, echocardiography, CMR, device-based follow-up
Thromboembolism/stroke	Cerebral embolic events, often in association with AF/AFL or atrial standstill	Permanent disability, recurrent events, increased mortality	Disproportionately high risk relative to patient age; requires proactive prevention	Rhythm surveillance, atrial mechanical assessment, anticoagulation-oriented evaluation

**Table 4:** Imaging phenotype in EDMD: echocardiographic and CMR findings with clinical interpretation. Echocardiography and cardiac magnetic resonance provide complementary information in EDMD, ranging from chamber remodeling and ventricular dysfunction to early myocardial deformation abnormalities and tissue characterization. The table highlights the incremental value of multimodality imaging for surveillance and risk stratification. Abbreviations: CMR, cardiac magnetic resonance; E/A ratio, ratio of early (E) to late (A) transmitral diastolic filling velocities; ECV, extracellular volume; EDMD, Emery–Dreifuss muscular dystrophy; HF, heart failure; LGE, late gadolinium enhancement; and LV, left ventricle/left ventricular.

Imaging Modality/Parameter	Main Findings in EDMD	Stage of Disease in Which Useful	Potential Clinical Value	Main Limitations
Standard transthoracic echocardiography	Atrial enlargement, variable LV systolic dysfunction, occasional dilated cardiomyopathy phenotype, chamber remodeling	Baseline and longitudinal follow-up across all stages	First-line structural and functional surveillance; identifies progression toward cardiomyopathy and HF	May underestimate early myocardial disease or subtle tissue abnormalities
Conventional Doppler/diastolic assessment	Impaired relaxation, altered E/A ratio, prolonged isovolumetric relaxation time, restrictive filling in advanced disease	Early subclinical dysfunction to overt cardiomyopathy	Detects early functional abnormalities beyond gross chamber dimensions	Interpretation may be affected by rhythm status, especially AF/AFL
Tissue Doppler imaging	Abnormal myocardial velocities despite preserved conventional indices in some patients	Early/subclinical stages	May reveal early myocardial dysfunction before overt LV remodeling	Operator-dependent and not fully standardized across centers
Speckle-tracking echocardiography/strain	Reduced deformation indices, impaired longitudinal mechanics, possible increased mechanical dispersion	Subclinical and intermediate disease stages	May refine early phenotyping and help identify patients at higher arrhythmic risk	Limited availability and limited EDMD-specific validation
Cardiac magnetic resonance: functional assessment	More accurate quantification of chamber volumes and ventricular function; detection of regional abnormalities	Particularly useful when echo findings are equivocal or disease progression is suspected	Improves phenotype definition and longitudinal characterization	Access and expertise may be limited
Cardiac magnetic resonance: tissue characterization	Mid-wall or septal LGE, increased extracellular volume, abnormal mapping indices, evidence of fibrosis substrate	Early to advanced disease, especially in *LMNA*-related phenotypes	Supports arrhythmic risk refinement and identification of subclinical myocardial involvement	Evidence in EDMD-specific cohorts remains limited and partly extrapolated from laminopathy series
Combined imaging approach	Echo for serial surveillance plus CMR for phenotypic clarification and tissue characterization	Across the disease continuum	Best overall strategy for integrated follow-up and risk assessment	Resource-dependent and not always feasible in routine practice

**Table 5:** Pragmatic management of cardiac complications in EDMD: surveillance, anticoagulation, device therapy and advanced options. The table proposes a clinically oriented framework for the surveillance and treatment of the main cardiac scenarios encountered in EDMD, from early asymptomatic stages to advanced arrhythmic disease and heart failure. Particular emphasis is placed on proactive rhythm surveillance, anticoagulation when atrial disease emerges, and the individualized selection of pacing versus defibrillator strategies. Abbreviations: AF, atrial fibrillation; AFL, atrial flutter; AV, atrioventricular; CMR, cardiac magnetic resonance; CRT, cardiac resynchronization therapy; ECG, electrocardiogram/electrocardiography; EDMD, Emery–Dreifuss muscular dystrophy; HF, heart failure; ICD, implantable cardioverter–defibrillator; *LMNA*, lamin A/C gene; LV, left ventricle/left ventricular; NSVT, non-sustained ventricular tachycardia; VT, ventricular tachycardia; and VF, ventricular fibrillation.

Clinical Scenario	Main Problem	Suggested Management Approach	Key Caveats in EDMD	Escalation Options
Asymptomatic genotype-positive patient/early phenotype	Silent progression of conduction disease, atrial disease, or myocardial involvement	Lifelong structured surveillance with ECG, prolonged Holter, echocardiography, and CMR when available	Cardiac manifestations may precede or outweigh skeletal symptoms	Earlier referral to inherited cardiomyopathy/neuromuscular center
Progressive conduction disease/bradyarrhythmias	Symptomatic bradycardia, advanced AV block, chronotropic incompetence	Permanent pacing according to guideline-based indications	Pacemaker therapy does not abolish the risk of malignant ventricular arrhythmias or sudden death	Consider ICD instead of pacing alone in selected high-risk patients
AF/AFL	Tachyarrhythmia, symptoms, stroke risk, progression of atrial disease	Rate/rhythm control strategy plus anticoagulation when indicated	Brady–tachy syndrome and coexisting conduction disease are common	Intensified rhythm monitoring, device-guided surveillance
Atrial standstill	Severe atrial myopathy, absent atrial contraction, embolic risk, atrial lead failure/loss of capture	Individualized pacing strategy and strong consideration of anticoagulation	Represents advanced atrial disease and may complicate device management	Device revision, specialist electrophysiology input
Ventricular arrhythmias/high-risk arrhythmic phenotype	NSVT, sustained VT/VF, sudden death risk	ICD-oriented risk stratification, especially in *LMNA*-related disease or when additional high-risk markers are present	Ventricular arrhythmias may occur despite only mild or moderate LV dysfunction	ICD implantation, tertiary arrhythmia referral
LV dysfunction/heart failure	Progressive cardiomyopathy with symptomatic HF	Guideline-directed medical therapy, serial imaging, clinical reassessment, device optimization when appropriate	EDMD-specific HF evidence is limited; progression may be genotype-dependent	CRT consideration, advanced HF referral
Recurrent arrhythmias despite drug/device therapy	Recurrent ICD therapies, refractory flutter/VT, electrical instability	Selected catheter ablation in expert centers	Diffuse fibrosis, intramural/atrial substrate, and disease progression may reduce success rates	Repeat ablation, combined advanced device/HF strategy
End-stage disease	Medically refractory HF and/or severe arrhythmic burden	Evaluation for advanced heart failure therapies	Rare but clinically relevant in advanced EDMD	Heart transplantation

## Data Availability

No new data were created.

## Abbreviations

The following abbreviations are used in this manuscript:

A	late transmitral diastolic filling velocity
AF	atrial fibrillation
AFL	atrial flutter
AI	artificial intelligence
APBs	atrial premature beats
AT	atrial tachycardia
AV	atrioventricular
AVB	atrioventricular block
BMD	Becker muscular dystrophy
CK	creatine kinase
CMR	cardiac magnetic resonance
CRISPR/Cas	clustered regularly interspaced short palindromic repeats/CRISPR-associated system
CRT	cardiac resynchronization therapy
CTG	cytosine–thymine–guanine repeat expansion
DCM	dilated cardiomyopathy
DDD	dual-chamber paced, dual-chamber sensed, dual response to sensing
DMD	Duchenne muscular dystrophy
E	early transmitral diastolic filling velocity
ECG	electrocardiogram/electrocardiography
ECV	extracellular volume
EDMD	Emery–Dreifuss muscular dystrophy
EMD	emerin gene
EMG	electromyography
ESC	European Society of Cardiology
HF	heart failure
HRS	Heart Rhythm Society
HV	His-ventricular interval
ICD	implantable cardioverter–defibrillator
ICMJE	International Committee of Medical Journal Editors
iPSC	induced pluripotent stem cell
LGE	late gadolinium enhancement
LINC	linker of nucleoskeleton and cytoskeleton
LMNA	lamin A/C gene
LV	left ventricle/left ventricular
LVEF	left ventricular ejection fraction
LTVTA	life-threatening ventricular tachyarrhythmias
MACE	major adverse cardiovascular events
miRNA	microRNA
NDLVC	non-dilated left ventricular cardiomyopathy
NSVT	non-sustained ventricular tachycardia
NYHA	New York Heart Association
PM	pacemaker
RNA	ribonucleic acid
SCD	sudden cardiac death
SVT	supraventricular tachyarrhythmias
SYNE1	spectrin repeat containing nuclear envelope protein 1 gene
SYNE2	spectrin repeat containing nuclear envelope protein 2 gene
TGFβ	transforming growth factor beta
VA	ventricular arrhythmias
VF	ventricular fibrillation
VVI	ventricular paced, ventricular sensed, inhibited response
VT	ventricular tachycardia
